# Caregivers’ experiences of providing care to female patients in Swedish forensic psychiatric care

**DOI:** 10.3389/fpsyt.2025.1646726

**Published:** 2025-11-05

**Authors:** Jessica Revelj, Ulrica Hörberg, Märta Wallinius, Mikael Rask

**Affiliations:** ^1^ Department of Health and Caring Science, Linnaeus University, Växjö, Sweden; ^2^ Department of Research, Regional Forensic Psychiatric Clinic, Region Kronoberg, Växjö, Sweden; ^3^ Center of Ethics, Law and Mental Health, Institute of Neuroscience and Psychology, The Sahlgrenska Academy, University of Gothenburg, Gothenburg, Sweden; ^4^ Evidence-based Forensic Psychiatry, Department of Clinical Science, Lund University, Lund, Sweden

**Keywords:** women, forensic psychiatry, coercive measures, care relationship, care environment, female patient, phenomenological hermeneutical method

## Abstract

**Introduction:**

A core element of caring science, is for caregivers to understand and collaborate with patients. Previous research shows that women receiving forensic psychiatric care evoke strong emotions among caregivers, but there is little research on the subject, and increased knowledge about caregivers’ experiences of providing care for female patients in forensic psychiatry, and of balancing healthcare with public safety considerations, is needed for the continued development of evidence-based care.

**Method:**

This qualitative study aimed to describe and gain a greater understanding of caregivers’ experiences of providing care for female patients in forensic psychiatric care. The study is based on 14 interviews with caregivers working with female patients at four forensic psychiatric clinics. The data were analyzed using a phenomenological hermeneutical method, which is suitable for researching lived experiences. The analysis was conducted in accordance with the method described by Lindseth and Norberg and revealed that caregivers emphasized the importance of being present in relation to the women.

**Results:**

The following themes emerged: *Providing care that is responsive to the conditions in forensic psychiatric care, Being an individual in a caring relationship, Providing support and strength, and Allowing the patient’s needs to determine the course of the care*. The women were perceived as less physically strong than male patients but engaged in acts of violence against themselves and others. This could result in coercive measures that could also be experienced as uncomfortable for the caregivers, particularly when having to restrain the women.

## 1 Introduction

Caring science focuses on the holistic aspects of a person’s health (1), and it is essential from this perspective to understand how caregivers experience patients’ suffering, health, and pathways to increased well-being, as well as how caregivers perceive the caring and care environment (2). De Vogel and Louppen stated that stronger feelings are evoked in caregivers by female patients than by male patients, and they perceive women as more deceptive and demanding (3). The authors report that caregivers exhibit more positive feelings (accepting, helpful, relaxed, and receptive) towards female patients compared to male patients, whom the caregivers perceive as more anxious, threatening, and overwhelming (3). Emotional responses, such as those described above, among caregivers may affect the care relationship and hinder effective treatment (2). It is therefore essential that caregivers process the potentially negative feelings that arise while providing care for patients (2). Female patients may give rise to diverse feelings among caregivers due to the women’s vulnerability, visible psychiatric and emotional issues, and their longing for help (4).

Female patients constitute a minority group in forensic psychiatry (3). The Swedish National Board of Health and Welfare reported that only 197 (13%) female patients received Forensic Psychiatric Care (FPC) in 2023, after having been sentenced to it by a court (5). People in Sweden who have been convicted of a crime may be subject to a forensic psychiatric assessment, which is conducted in pre-trial custody. If they are assessed as suffering from a severe mental disorder, they are still, according to the Swedish legal system, viewed as responsible for their criminal actions and can be sentenced to FPC, a legal sanction among others (6). The caregivers must follow the Health and Medical Services Act (2017:30) when providing care to the patients. However, FPC is also regulated by supplementary laws: The Compulsory Mental Care Act (1991:1128) and the Forensic Mental Care Act (1991:1129). Possible coercive measures are regulated in the first of these laws, and the use of different security levels to provide care for people suffering from severe mental disorders are emphasized in the second. FPC presents clinical, juridical, and ethical challenges for caregivers due to the fragility and vulnerability caused by the patients’ severe mental disorders (7). The patient group (both male and female) is vulnerable and faces precarious situations, partly due to their illness and the index crimes, and partly due to the institutional environment characterized by a high level of security where they are cared for against their will (8, 9). The wards are organized according to different security classifications: the very high security level (I) is equipped with a perimeter and structural protections capable of resisting escape attempts, including those involving assistance; the high security level (II) must be able to withstand escape attempts and the satisfactory security level (III) includes operational routines for monitoring patients’ whereabouts (10). Sollied and Kvande described that the environment with structure and predictability was proper and created an atmosphere beneficial for the patients’ disorders to become less prominent (7). Providing care for patients in such settings can be challenging, and it may take years of experience and practice to become an effective caregiver (3). The combination of the patients’ history of serious offending and complex psychopathology is demanding and requires caregivers to be highly skilled (2). Caregivers need to provide humanistic care, despite being aware of, yet not influenced by, the patients’ difficult life circumstances (8). The most common crimes committed by female patients were, according to the Swedish National Forensic Psychiatric Register (11), crimes against life and health. Previous research indicates that comorbidity is common and that female patients, when compared to male patients, seem to present a different psychopathological panorama with more depression, borderline personality disorder, and post-traumatic stress disorder but less antisocial personality disorder (4). They also seem to have a complex history of victimization with higher rates of sexual abuse, more self-harm and suicidality, and their offenses are more often directed towards family and children (4). The women seemed to exhibit a behavior where they claimed to be pregnant, were seriously ill, or falsely accused male patients or caregivers of harassment, which was less common among male patients (ibid).

Female patients who receive care in Swedish FPC are mostly treated on the same wards as their male counterparts (12). Female patients have, throughout the years, received care on wards with high security due to aggressive behavior against others, and in some cases, when general psychiatry has been unable to reduce severe self-harm during the care, or when treatment on wards with lower security levels has been logistically impossible (13, 14). Providing therapy for female patients in FPC requires an understanding of their life situation. It has been proposed that these women have particular needs, challenges, and vulnerabilities (12, 15); however, there is a knowledge gap concerning these needs (16). Caregivers must become familiar with each patient’s individual life to understand how they cope with and carry their own narratives, history, and experiences, as well as how they interpret their surroundings and the intentions of others. In another study (6), nurses were helped by being aware of and understanding the patients’ needs in their daily lives when caregivers spent time with and involved the patients in decision-making (7). It has been demonstrated that caregiver-patient interactions play a crucial role in the care relationship (16). Caregivers are considered essential for being supportive and open to female patients’ narratives by listening to their experiences, acknowledging their emotions, and taking them seriously (17). The authors states that several factors, such as compulsory care and the restricted environment that characterizes FPC, are essential for the health and experiences of female patients. This could also subsequently affect the caregivers’ experiences of providing care to the female patients. Alternatively, one could reverse the perspective: what caregivers’ experience in relation to female patients and FPC could affect the care they provide for female patients. Research on female patients and their caregivers in FPC in Sweden is sparse, and further studies are needed (18). The caregivers’ situation in relation to the female patients and the care provided can be highlighted by studying the caregivers’ experiences of female patients in FPC, thus generating crucial knowledge for the much-needed continued development of FPC (19). Studies on female patients and their perception can contribute to a better understanding of how and what care to provide for these patients to improve their health.

## 2 Aim

This study aimed to describe and gain a greater understanding of caregivers’ experiences of providing care to female patients in forensic psychiatric care.

## 3 Methods

This qualitative study is grounded in a phenomenological hermeneutical approach, inspired by Ricoeur (20). When focusing on caring science and lifeworld, it could be seen as essential to use a method where someone with lived experience shares their history and knowledge. The focus in this study is on caregivers’ experiences of providing care to female patients; however, in the long run, even the female patients themselves may be impacted by the caregiver’s approach to care. A description of a phenomenon (providing care to female patients in FPC) was sought through a phenomenological approach. While an interpretation of what appears in the caregivers’ narratives was sought in accordance with hermeneutics, both were based on the caregivers’ lifeworld (21). The authors were aware of and tried to control their preunderstanding during the phenomenological part to allow the content to appear (22). The analysis has been performed using a method described by Lindseth and Norberg (23, 24), where the importance of following the movement of the text and tracing the progression from what is said to what is revealed is emphasized (23).

### 3.1 Procedure

The managers of four (randomly chosen) forensic psychiatric clinics approved the study to be conducted at their hospital, whereafter ward managers permitted the researchers to inform the ward staff (caregivers) about the study and request their written consent for participation. There was one clinic that did not allow the authors to ask the employee for participation, but instead provided suggestions for specific employees to approach for participation; this clinic did not participate in the study. The initial written information about the study was sent to prospective participants via email by the first author. The same information was then provided to the participants, both verbally and in writing, prior to the interview and all participants signed an informed consent prior to commencing the interviews. Their participation was voluntary, confidential, and they were able to interrupt the interviews or withdraw their consent at any time without providing any reason. Data were collected from interviews with 14 caregivers (six men and eight women) between April 2022 and December 2023. The interviews were individual and semi-structured, allowing participants to narrate what they found most important in their experiences of providing care for female FPC patients. Nine interviews were conducted in person, and five were conducted digitally via video calls. The following initial questions were asked in all the interviews: “What in the care provided for female patients do you consider the most distinctive?” “In what way do you think the female patients are affected by the care environment?”. Follow-up questions based on the participants’ responses were also posed. The interviews were audio recorded and transcribed verbatim; names and places were changed so that the narratives could not be linked to a specific person. The interviews lasted between 17 and 47 minutes (M = 32) and were conducted at a time and place chosen in consultation with the first author and each participant.

### 3.2 Participants

The inclusion criteria were caregivers (who, in the following text, refer to salaried nurses, assistant nurses, and social workers) who had worked with female patients on a FPC ward for at least six months over the last two years. To the author’s knowledge, caregivers have not received specific additional training to care for female patients. A few nurses had specialized education in psychiatric care, and the nurse assistants had also received some education on psychiatric care. Six men and eight women participated; nine of them were registered nurses, and five were assistant nurses or social workers. The ages of the participants ranged from 25 to 62 years (M = 41), and their length of employment on a FPC ward varied from 1 to 34 years (M = 9.6). All the participants spoke Swedish, with most of them using it as their first language. The participants worked in care settings with varying security levels: satisfactory (Level III), high (Level II), and very high (Level I).

### 3.3 Analysis

The interviews were transcribed verbatim, and the analysis was conducted in accordance with a method described by Lindseth and Norberg (23, 24). This analysis consists of three steps, the first step of which involves reading the assembled material several times to identify the prominent content. This is termed the naïve reading and is seen in the manuscript as the naïve understanding, describing the phenomenon of providing care to female patients in FPC as a whole. The structural analysis (second step) methodically explains the analyzed text in relation to the phenomenon. Meaning units, comprising several sentences, were condensed to smaller units (**Table 1**). These units were compared and analyzed for differences and similarities, resulting in 12 subthemes (**Table 2**). This step was conducted four times to ensure that the subthemes could stand alone while also being part of the whole text. Groups of subthemes were created, resulting in four themes (**Table 2**) and it was determined whether these themes reflected the naïve understanding formed in step one. The third and final step entails reading the material as a whole with an open mind and with the preunderstanding under control. The results were then examined in relation to the research question, the study’s context, and previous research (23), which is crucial for highlighting the results within a broader context. The first author performed an initial analysis, which was then validated by the second and fourth authors. This process was carried out manually without the use of software.

**Table 1:** Structural analysis: examples from caregivers’ experiences of providing care to female patients in FPC.

Structural analysis			
Meaning units	Condensation	Subtheme	Theme
It also creates frustration in a way, I think, among the staff, who sometimes find it difficult … I would say it’s involuntary … They feel like they’ve broken an alliance of trust or something like that when, for example … well, when staff members say they have a very good relationship with a patient, and then they almost take it as, excuse the expression, a personal insult or betrayal if something bad happens.	It evokes involuntary frustration among the staff when they feel that an alliance of trust has been broken. For example, if staff members say they have a good relationship with a patient, they take it as a personal insult or betrayal if the patient gets hurt.	Managing vulnerability and providing care based on psychosocial conditions	Providing care that is responsive to the conditions in forensic psychiatric care
Many of them have not … um, how should I put it, in some normative way, lived as women in their lives. I mean, they’ve been different early on, made other life choices, and perhaps haven’t prioritized having a stable partner, having children, or focusing on family. They may have stopped expecting that early on. Instead, it’s the family that has taken care of them.	Few of them have lived as women from a normative perspective. They have been different early on, made other choices, and haven’t prioritized having a stable partner or having children. Their families may have stopped expecting that early on, and instead, the family has taken care of them.	Recognizing and providing support in relation to the contextual needs of the patient	Providing support and strength

**Table 2:** Themes and subthemes.

Caregivers’ experiences of providing care to female patients in swedish forensic psychiatric care					
Theme	Subtheme				
Providing care that is responsive to the conditions in forensic psychiatric care	Providing care based on the physical and structural conditions in the care environment	Managing vulnerability and providing care based on psychosocial conditions	Being able to see and respond to vulnerability		
Being an individual in a caring relationship	Interacting with the women in the care relationship	Recognizing the significance of identity in the caring relationship			
Providing support and strength	Creating structure, meaning, and empowerment	Recognizing and providing support in relation to the contextual needs of the patient	Supporting healthy relationships	Providing support for the role of being a mother	Managing another person’s vulnerability and right to sexuality
Allowing the patient’s needs to determine the course of the care	Seeing the female patient in the forensic psychiatric care process	Encountering physical and emotional expressions and allowing needs to dictate (safety) precautions			

## 4 Results

### 4.1 Naïve understanding

The caregivers stated it was less common to care for female patients in FPC compared to male patients, and the women were seen as severely affected by their mental disorders and previous experiences. It was described as essential to see the women as individuals for whom the caregivers needed to create a safe ward, even though the language on the ward could be coarse. The caregivers were sometimes unable to see whether the female patients were being exploited, e.g., sexually. They needed to be protective of the female patients, and it was perceived as being difficult for the female patients to maintain their femininity, e.g., due to not being able to provide suitable clothes.

The care relationship between female caregivers and female patients could be either sisterly or one in which female caregivers place greater demands on female patients than on male patients. It was also noted that female patients were physically aggressive toward female caregivers to a greater extent than towards male caregivers. The latter were concerned that female patients might misinterpret their intentions, so they ensured a coworker accompanied them to minimize the risk of the women feeling exposed in the care situation. Established and lasting contacts were beneficial, and caregivers adapted their behavior, for example, by trying to be more accommodating, adopting a gentler approach, and expressing their requests more carefully or differently than they would for male patients. They were also more prepared in conversations when providing care to female patients. The caregivers tried to motivate the female patients to maintain contact with their relatives. However, they were also aware that in some cases the relatives did not always have a prosocial influence on the female patients, or that the relatives were victims of the female patients’ offences.

They experienced that female patients were allowed to participate in various activities or were reinstated relatively quickly if activities had been withdrawn. It was sometimes difficult to motivate the women to participate despite a wide range of activities. The caregivers noted that they had less focus on possible violence or deviant behavior from the female patients than with male patients during activities outside the hospital, such as walks, or when the women were expected to receive negative information. Caregivers chosen to interact with the women in situations where outbursts were expected had less physical strength compared to those in the same situation with male patients. The female patients were seen as less capable than male patients of serious violence during the care process, regardless of whether the index crime was more serious. The female patients were perceived as planning their way of acting out and had a clear focus on a specific outcome or person. It was common for women to use various bodily fluids, e.g., menstrual blood, urine, or feces, during outbursts of aggression.

It was easier for caregivers to perceive the women’s vulnerability if they needed help or when their mental health had deteriorated, compared to male patients. This was assumed to be due to the women using various tools to communicate their feelings, e.g., through self-harming behavior. Coercive measures could be avoided if caregivers were aware of and recognized the signs of impaired health. The caregivers experienced that female patients were more often subjected to coercive measures and received a higher level of supervision more often in comparison to male patients, but these actions were usually only short-term. It was uncomfortable for male caregivers to perform coercive measures because they perceived it as an assault on the female patient.

### 4.2 Providing care that is responsive to the conditions in forensic psychiatric care

#### 4.2.1 Providing care based on the physical and structural conditions in the care environment

Caregivers described the care environment as controlling and restrictive, where activities are limited to areas where the women need to be accompanied by caregivers. On some occasions, they need to prevent the women from performing everyday tasks due to the ward’s cleaning and hygiene routines. Behaviors and belongings viewed as usual from a societal perspective could be perceived as abnormal or dangerous within the care environment, which affects the ability to support the women to maintain their femininity. It was possible to assist patients with a clipper when cutting their hair, which was not seen as accommodating to women’s needs. Caregivers felt that they were unable to provide patients with appropriate clothing, as the clothes were often transparent, poorly fitted, and rarely served the intended purpose of the garment. The wards were prepared to provide necessary hygiene products for men, but it was difficult to obtain items such as sanitary pads or hairbrushes when the female patients needed them.

*“There’s no suitable underwear … it feels as if a woman has big breasts, for example … and then they don’t have their own clothes, then they have to wear a big t-shirt that is sort of transparent, and you need to find ways to help them cover up”* (Female caregiver, 39 years)

The caregivers secured the women’s rooms because of a fear of suicide by removing belongings considered dangerous or that were not permitted on the ward. Some women were not allowed to wear bras, as this could be a potential object for self-harm or harm to others. However, caregivers thought that removing personal belongings could influence the women in managing their mental health, as personal belongings could motivate and help them cope with emotional distress.

The caregivers noted that the women avoided areas where the architecture provided little privacy and “hidden” spaces, as these could expose them to danger from other individuals. The caregivers wanted to protect the women and accommodated them in rooms with good visibility or where another woman lived next door. However, these aspects were only considered in heterosexual constellations, and it was not always certain that women appreciated the presence of other women, and the placement became disadvantageous.

*“It would probably be more considerate if those of the same gender, especially when women who are a minority on the ward, wanted to be closer to each other, then it would have been possible just for practical reasons if, for example, there was a shower or shared toilet, but we wouldn’t have moved a man and a woman to the same part of the ward just so they could be close to each other”* (Female caregiver, 35 years)

#### 4.2.2 Managing vulnerability and providing care based on psychosocial conditions

Female patients were not commonly encountered in FPC, but differentiated wards were not considered advantageous. The caregivers did not want to make distinctions between genders and intended to treat each patient individually, regardless of gender. They noted that the structure of the care was designed for male patients, with the women and their needs placed more in the background. The caregivers aimed to create a secure environment for the women and considered themselves to be gentle in their approach. However, they acknowledged that this behavior could vary depending on a specific female patient and not all female patients as a group. The caregivers were aware that the FPC context could affect the women. Still, they needed to assess each woman’s individual interests, behavior, and personality, and treat each woman, regardless of gender, with creativity to maintain normality.

*“People are in some way the sum of their experiences … but it’s clear that if you’re in a context where you’re rarely treated in a certain way, you have certain expectations of yourself, you will adapt and then it becomes your personality and your individual … individual … individuality or something like that, although it may be shaped by society”* (Female caregiver, 34 years)

The women were perceived as being severely affected by their illness, and the caregivers thought there could be a higher threshold for women to be sentenced to FPC. They witnessed and were concerned about the women’s outbursts and self-harming behavior, despite perceiving them as less dangerous. They attempted to moderate the female patients’ emotions. They were open to involving them in the care process, offering various treatments (although still limited) if the previous one had been unsuccessful. They experienced the psychiatrists as being more lenient in their assessments, allowing autonomy to a greater extent compared to the male patients. The length of care was advantageous, allowing for long-term observations of behavior and actions from different perspectives and the testing of various interventions over time. FPC, with its facilities and caregivers, was a permanent feature even in the event of an unsuccessful leave of absence or relapse into substance abuse. Established contacts were experienced as beneficial, as they became familiar with the women and were accessible, allowing for the evaluation of interventions in a reliable environment.

*“A stable place that is always there if you fail when on leave, if you fail on … yes, yes, but if you relapse into abuse or whatever it could be, there’s a place that’s always there … which does not judge whether you need inpatient care or not”* (Male caregiver, 39 years)

The women spent a lot of time in their rooms, thus making it difficult to establish a relationship, although the caregivers felt that the women wanted contact, answers, and explanations. The women tended to categorize caregivers as either good or bad, which created dissension among the caregivers. They emphasized the importance of giving positive feedback during occasions when the women had difficulties and of showing their curiosity, referring to positive moments without engaging in self-harming behavior. After a period when the women’s mental health had deteriorated, the caregivers worked together with the women to form a course of action that could be used on the next occasion the women’s mental health was impaired. There was, however, a risk of making positive interpretations of the women’s well-being, leading to assumptions that the absence of self-harming behavior meant the women were doing well even if that was not the case.

#### 4.2.3 Being able to see and respond to vulnerability

The caregivers noted that non-differentiated care increased the vulnerability of the women, which led to heightened levels of vigilance regarding the women’s safety. They attempted to protect the women from vulnerability and found it possible to address the women’s emotional expressions when they appeared to be in touch with their emotions. The caregivers found it easier to recognize when they had not provided adequate support for the women, as the women tended to express their emotional state more clearly than men, e.g., their suffering could be observed through the women’s self-destructive behaviors.

*“I’d like to say that it’s just a way of saying that I’m still not feeling well, so that I should start with the polite I’m feeling well or should I show that I’m not feeling well, then I think it’s better to signal with the only tools one has, unfortunately”* (Male caregiver, 39 years)

The caregivers who participated in the study described a common misconception among caregivers that self-harming behavior should be responded to with noninterference. They instead believed they should be interested and inquisitive about women’s emotional state. They had witnessed more self-harming behavior among women in the past. They related this to differentiated wards where the women’s emotional state seemed to have a greater influence on the other female patients on the ward.

### 4.3 Being an individual in a caring relationship

#### 4.3.1 Interacting with the women in the care relationship

The women were described as challenging to manage, seeking positive feedback and validation. The caregivers did not want to experience fear when working with female patients, but they required being prepared to learn about themselves as individuals and in their professional role. They were not unanimous in their approach to the women; some spoke of being more cautious and choosing their words carefully, while others set boundaries early on. They expressed good intentions in their approach, but felt their interactions and striving for good, caring relationships were complex. They listened to the women’s experiences and shared their own stories with the women. It was seen as the caregiver’s responsibility to build trust that helped them understand the women’s anxiety so that they could observe symptoms. Trust seemed to increase when they were truthful, provided honest information, acknowledged the situation, and met the women’s needs. Caregivers believed that the women should be able to trust them and that they managed to find what motivated the women. The interaction could be reflected in women’s personalities, as well as when caregivers became distant and set stricter boundaries early on.

*“just the way you … deal with … this sometimes rather harsh, joking language in order to meet the macho guys might not go down well with some of the female patients who I understand*, at least on our ward, are a little more cautious than many of the men are” (Female caregiver, 25 years)

On a few occasions, the caregivers understood the women’s sadness and somatic complaints as a way of creating drama. The caregivers could be so involved in care situations that it posed a risk of affecting other responsibilities. If they experienced a good relationship with a specific woman, it could be experienced as a betrayal and personal insult if she harmed herself. Some caregivers adopted a protective role, whereas others responded with avoidance, distancing themselves from female patients and other caregivers, particularly in situations when the woman displayed sadness or was perceived as demanding. This behavior was contrasted by the caregivers with the response to male patients, whose use of threats and violence often led caregivers to acquiesce. Despite these contrasting strategies, the majority of caregivers reported striving to approach female patients in the same manner as other patients, aiming to remain neutral and non-judgmental in their interactions.

#### 4.3.2 Recognizing the significance of identity in the caring relationship

The caregivers had mixed experiences of how their gender influenced the women and the caring relationship. Sharing personal experiences and navigating the balance between being private and being professional was considered beneficial, but not always easy. Female caregivers could (according to both male and female caregivers) be stricter towards the female patients, which was believed to stem from an expectation of different capabilities in the women receiving care. A caring relationship involving female caregivers and female patients reduced the risk of the relationship being misinterpreted as inappropriate or misunderstood. The female caregivers thus found physical contact, such as holding hands, less problematic. Female caregivers noted that their maternal role could facilitate caregiving as they were accustomed to managing conflicts, giving reprimands, and promoting others to maintain good hygiene. The caring relationship could be based on shared interests, and perceived as sisterly or friendship-based, but sometimes they demonstrated a stricter approach than their male colleagues.

*“Yes, it is, it’s hard to work with women. But also a lot, a lot of fun, like woman to woman, even if I even if I get kicked and threatened and so on, it’s usually great fun, it’s really great fun working like this, ohh, I’m going to buy this dress, I’m going to get this from my trustee, like, you play bingo together, you have a different connection”* (Female caregiver, 29 years)

The women were seen to prefer speaking to female caregivers when it came to hygiene or sexuality concerns. However, gender was less relevant when fulfilling requests, and in some cases, male caregivers were perceived as more accommodating. The ability to approach women may be influenced by cultural heritage, professional title, and the age of the caregivers, with younger caregivers being perceived as humbler. Male caregivers found it problematic and were concerned about whether the correct approach was used when they had to perform medical procedures requiring women to remove garments. They ensured they were accompanied by other caregivers, especially when female patients filed complaints about caregivers they deemed inappropriate. They were also aware that the words and tone of voice could influence the woman’s interpretation due to the caregiver’s gender, which made them extra cautious.

*“I’d probably say that I think it’s the men themselves who do it, who take the initiative for it … that they’re quite careful not to be alone in the room or … and sort of make a plan around it even if there aren’t any guidelines or anything about it”* (Female caregiver, 39 years)

### 4.4 Providing support and strength

#### 4.4.1 Creating structure, meaning, and empowerment

The caregivers experienced that some women were placed in lower-security wards sooner than men in similar situations, despite demonstrating overt physical aggression. In other cases, there was no difference in the progression of the care process. It was described as essential to strengthen women’s identity and ensure that meaningful activities were tailored to their interests. However, there seemed to be a normative division, where women were invited to activities such as sewing or embroidery to a greater extent, but less frequently to tasks like heavy gardening work. The caregivers found it challenging to help women maintain their femininity and saw it as essential to protect them from harassment. The caregivers perceived that the women appeared indifferent when receiving negative information or during events that are generally difficult to process (e.g., abortion). The caregivers believed this could be due to the women having experienced more severe or traumatic events earlier in life.

*“So their bar for what’s difficult and what they expect from life is low, oh it is, oh it is … You can also have to work with that, it’s what it is, I know during corona, yes, but it’s not normal that you should be isolated for 3 weeks because someone had corona in the ward next door, but then they’re like … I’ve been through worse. I was held in custody for 6 months”* (Female caregiver, 39 years)

#### 4.4.2 Recognizing and providing support in relation to the contextual needs of the patient

The female patients initially appeared to ally with one another; however, over time, their relationships began to taper off, and they preferred to interact with the caregivers. This happened even though the caregivers could sense the women distancing themselves from them, as they believed the women viewed them as guards. The caregivers observed, however, that the women tended to adopt self-harming behavior and acts of violence towards others, which caused concern among the caregivers. They believed the women had not prioritized finding a partner or having children in their life choices. They might have been cared for by their families well into adulthood. Being in FPC was seen as an opportunity; the women were more alike and able to find a sense of belonging, as fewer had families, children, or other characteristics typically expected of women in general.

*“It’s a little harder to be a woman in society, and it’s almost easier here, because there’s no one who has a family and children here, and is especially, what can I say … Who has all the qualities that you can be expected to have as a woman; be able to take care of others, be able to take care of oneself or be able to take care of one’s appearance, one’s hygiene, things like that, they become a little more, yes, a little more like each other”* (Female caregiver, 39 years)

If the women used less violence, they could return to their home county before others on the waiting list. At the same time, caregivers encountered women with unrealistic expectations regarding the care process and future housing conditions. It can be challenging to find suitable accommodation, as there are generally fewer women in institutions; however, caregivers felt that the care facilities were welcoming towards the women. The caregivers stated that being a female patient outside the hospital could be experienced as complex and unfamiliar, leading to situations that could interrupt the care process.

*“When it starts to get close, it’s so classical that it becomes dangerous, almost a threatening situation, so you almost ruin it for yourself, because here you know what you have … Out there you don’t know”* (Female caregiver, 29 years)

#### 4.4.3 Supporting healthy relationships

The women had few relatives, which made them more likely to socialize primarily with other patients and caregivers. This became evident and was perceived by the caregivers as a need for companionship, protection, or other benefits. The women may have lost relatives as a result of their crime, either as victims of the crime or because the family distanced themselves afterwards. Having a broken or non-existent social network was identified as a risk in relation to providing better health. The women formed new and, in some cases, destructive social networks during the care. Facing two destructive social networks rather than one was something the caregivers needed to manage to support the women. It was essential for them to listen attentively to the women and acknowledge their diverse needs for social interactions, as interpersonal relationships were seen as a human need.

*“It’s natural to want different types of relationships, but the patients basically only have each other so they need to develop a companionship or bond with each other, and then just something like a physical touch, or closeness. Even in a non-sexual way, I think this is a fundamental human need”* (Female caregiver, 34 years)

The caregivers attempted to foster good contact with relatives and facilitated both indoor and outdoor visits. However, these meetings could be hindered by the relatives’ willingness or unwillingness to maintain a relationship with the woman. The caregivers witnessed positive relationships where contact had a positive impact on the women’s well-being, but they also witnessed destructive family relationships.

*“…but the mother embraces her daughter, she (the female patient, author’s remark) shouldn’t have help, medicine, or agree to anything we think she should do, at the same time the daughter wants to receive help and medicine so she is very split, if she talks to her mother on the phone, she may feel bad afterwards, gets angry, screams and stuff, and then it affects us”* (Male caregiver, 58 years)

The caregivers found it beneficial for the women to have a structured schedule with predetermined times for contact with relatives, thereby reducing excessive and repetitive contact with authorities or relatives, which had adverse effects on the women.

#### 4.4.4 Providing support for the role of being a mother

Being a mother seemed to unify some of the female patients. Still, the involvement of children led to discussions among the caregivers, particularly when the children were physically and psychologically distant from their mothers. The caregivers encouraged contact even though this could be complicated by families who did not want the children to visit their mothers, or when the women no longer had custody of their children. The caregivers expressed concern about what the children had seen or experienced in relation to their mother and considered the potential harm the mothers could cause or whether the child or female patient would become negatively affected by the contact.

*“There’s a fear they might even hurt their children, or disappoint them I think, might be the biggest thing and ehhh like that. I mean that she might feel bad and not able to care because of not having met her children, it’s clear that she ehh will be very sad for a while afterwards”* (Female caregiver, 29 years)

The caregivers reflected on whether it was best for the women to maintain contact with their children. They considered conversations about family and the role of parenthood to be common topics of discussion. Despite this, they observed little motivation from the women to maintain contact with or meet their children, particularly when the women became discharged and their behavior or craving for alcohol or drugs took precedence.

*We have someone who has children, eh, but has a highly egocentric mindset … it’s just her and her needs, I mean … she’d like it to look as though it’s the children first and foremost, but really what she’s thinking about is cigarettes she wants to buy instead”* (Female caregiver, 44 years)

#### 4.4.5 Managing another person’s vulnerability and right to sexuality

The caregivers stated that the female patients had a complex history, including substance abuse, mental illness, and, in some cases, prostitution. They encountered women who had used prostitution as an economic strategy that could be sustained by electronic communication and social media. This was seen as a form of affirmation, allowing the women to be a part of a community, albeit a destructive one. The caregivers perceived this behavior as more serious than the women seemed to be doing. They found this behavior challenging as they were unsure how to address recurring violence, prostitution, and vulnerability. They lacked information about the behavior, as the women viewed it as a possible source of income, to gain benefits, or something that negatively affected the care process. The caregivers wanted to be protective and emphasized that established caring relationships increased the likelihood of the women sharing information about their vulnerabilities. It was difficult for the caregivers to implement consequences for the possible perpetrator exploiting the women when the women were unwilling to cooperate.

*“No matter how much we want to protect women in forensic psychiatry, where they are a confirmed minority, it’s not enough. We have a lot to do, I wouldn’t say failed, but there is still a lot to do … and you also benefit from these long-term established relationships, I mean who do you want to tell if you have been exposed to something, uhhmm, I do that to someone I am comfortable with”* (Male caregiver, 39 years)

The caregivers needed to be supportive of the women’s interpretation of what the surroundings offered, helping them understand social codes, engage in verbal discussions, and provide assistance in resisting others’ suggestions of sexual intentions. This became more challenging when the women were on leave of absence. While being protective, the caregivers also had to balance information about sexual relationships, as such relationships could be initiated by the women as well, and everyone has the right to express feelings as long as both consent. However, they were more likely to consider relationships negatively when they involved heterosexual (primarily sexual) relationships, and they were less prepared for the potential negative consequences when two women established a relationship. Relationships and the women’s behavior could be based on desires for protection, cigarettes, and soda, leading them to adopt behavior promoting these outcomes, only to later disengage from the contact. There were thus occasions when caregivers needed to protect others from the women’s actions. In situations where the women’s mental disorders made it difficult to follow social codes, leading the women to expose themselves, the caregivers needed to be active and present to protect the women from making decisions they might regret later.

*“We had a patient who, uh, she had autism so she had these special interests and was super interested in a certain subject, and then she met a male patient who was also interested in the same thing and they talked a lot about this subject … then I know the caregivers were a bit like ahaaa but now they hang out a lot with each other, now we have to keep an eye on them so they don’t go into a room alone”* (Female caregiver, 34 years)

### 4.5 Allowing the patient’s needs to determine the course of the care

#### 4.5.1 Seeing the female patient in the forensic psychiatric care process

It was considered essential that treatments and diagnostic criteria were tailored to gender differences in behavior and symptoms in order to increase the likelihood of providing adequate care. The caregivers accommodated the women’s requests, possibly because they rarely damaged or threw furniture. They stated that the women conformed to existing approaches, but they needed to be vigilant to avoid being misled. According to caregivers, there was a historical tendency to view women as emotionally unstable and to project emotions onto caregivers in FPC.

*“There’s a perception among those who have worked for a long time, that someone has somehow gained or regained too many benefits a little too quickly. oh they think, their experience is that it’s because she’s a woman, she has somehow charmed or manipulated men in different ways and they see her as more innocent than she really is”* (Female caregiver, 34 years)

The caregivers intervened before the women’s behavior escalated, but were concerned about being deceived, as they saw the consequences of women’s violence as less harmful compared to the violence exhibited by men.

#### 4.5.2 Encountering physical and emotional expressions and allowing needs to dictate (safety) precautions

The caregivers perceived women as biologically less physically strong and threatening, with less fear of them committing serious violence, even though they could have daily violent outbursts. Fewer caregivers were needed to be involved in violent situations; however, they experienced the women as more demanding in terms of care, as they displayed their aggression in different ways. The caregivers mentioned that the women used menstrual blood, urine, feces, screaming, and other forms of behavior seen as self-degrading. The caregivers felt that when they encountered the women’s aggressive outbursts, these outbursts could have been planned over a more extended period and directed towards specific individuals.

*“it’s often pee and poo, and menstrual blood it could be … like boogers smeared on the walls and male patients can do that too but this was like a lot, ehhh. It feels like maybe a little more degrading like that, a man ahh peeing on himself or yes like being naked and running around”* (Male caregiver, 41 years)

Due to the expectations of less severe violence or fewer attempts to escape during supervised activities, less muscular and agile caregivers were chosen to care for the women in these situations. This approach was also applied when conveying negative information, but in the same situations, with male patients, they chose more muscular caregivers. The emotional expressions and aggressive behavior were directed towards the women themselves, other patients, or caregivers. Violence was seen as premeditated, and caregivers were assaulted when their attention was diverted. In cases of physical violence, women were subject to coercive measures for a shorter duration compared to men; interventions such as conversations, motivation, and increased supervision were often deemed sufficient. Early identification of deteriorating mental health was seen as essential to prevent escalation into self-harm. The caregivers noted that while women did not always meet the criteria for proportionate coercive measures, they were subjected to more of these actions than men. It was explained that women benefited from avoiding coercive measures, as this created a power imbalance.

*“they have these types of ehh behavior or self-harming behavior, which can be quite serious, but it’s clear that it’s not optimal, but although I think it might be beneficial, it might benefit the female patients not to be exposed to ehh, like most of the time, maybe men or like, it becomes like an imbalance, big men small girls”* (Female caregiver, 29 years)

It was experienced as difficult to use coercive measures against women, as it was easy to assume that women had previously been subjected to rape under restraint. Involvement of strong men during a restraint procedure was seen as an assault, where the women fought uncontrollably. Male caregivers described this action as constantly being extremely uncomfortable, as it felt like they were committing an assault.

*gets really dissociative sometimes and extremely self-harming, she bites herself, she chews off pieces of her arms … and then we need to restrain her, first hold her and then restrain her because it doesn’t stop … usually, who can handle it, yes it happens to be strong men aa so it feels like you’re standing there and raping her when you have to restrain her, it’s terrible every time. I never get used to it, I’ve been there several times, or been forced to be there several times and it’s ugh and then you have to pull down her pants because you have to give her a couple of injections and aa that’s it’s no, I never get used to it”* (Male caregiver 53 years)

The caregivers observed panic in the women’s eyes while using coercive measures such as restraints. To alleviate this, they stayed close to the restraint bed, offering reassurance and helping the woman shift focus. They found that restraining them for about 20 minutes was usually sufficient to help the women calm down.

## 5 Comprehensive understanding and discussion

Caregivers in FPC are expected to balance the needs for care, safety routines, and constraints in everyday life activities on the wards (25). Caregivers provide care tailored to the specific psychiatric needs, the environment, and the patient. The way caregivers approach their lifeworld will impact the lifeworld of female patients, at least during their time in the wards. However, at the same time, the lifeworld and actions of female patients will undoubtedly affect their caregivers. In caring science, it is essential to highlight someone’s lifeworld and how it is personalized; however, it is also crucial to keep in mind that it is not an entity in itself, but rather a part of a larger system. In this study, it has been highlighted that female patients influence caregivers in different ways and how they behave. Still, caregivers are also aware that their actions and presence affect the female patients. The results could be interpreted as describing the female patients’ behavior more than the caregivers’ experiences of providing care. It is thus essential to see that their behavior affects the caregivers and the way they provide care to the female patients. Furthermore, the caregivers’ behavior and care affect the women and the care environment.

Caregivers felt they were expected to comply with various guidelines, but at the same time, adapt to every single woman. Caregivers need to find a place of normality for each individual, which can be challenging (9). Differentiated wards were mentioned, despite being a minor subject in a few interviews, where caregivers noted that female patients were vulnerable; however, they all agreed on the advantages of mixed wards. This finding is consistent with a study of female patients (17). By using mixed wards, the female patients are often in the minority, and their possibilities to experience connection to another woman decrease. Female caregivers could therefore be a valuable resource for the female patients, e.g., how to be a mother, or how to dress and behave. It is essential to strike a balance in this type of identification. There is also a potential risk of identification with female patients, as stated in another study (26), and one needs to separate the idealizations that may be both flattering and frightening; this requires strategies for distancing oneself from these perceptions. Caregivers in the present study emphasized the importance of allowing female patients to experience some normality, such as listening to other women’s conversations. This could be an essential factor in female patients’ ability to identify with other women and perceive some normality. However, caregivers must also consider how their behavior and influence affect the women, and not be either flattered or intimidated.

The authors did not ask the participants if they had experience of providing care in different safety classifications; however, it is worth noting that different wards have varying possibilities, and there may, of course, be differences between safety classifications, clinics, and wards. While providing care to female patients, caregivers sometimes had to confiscate belongings, even though these could be important to the women in their everyday lives, which could affect the caregivers’ ability to support the women in preserving their femininity. This can be seen in a circular way, where the female patients’ needs and assumed dangerousness create a more austere environment. Such a minimalist environment could accustom the female patients to being without these belongings, potentially making it more difficult for them to acclimatize back to society. Would they then be prepared for a life in society? A similar mindset could be applied in caring relationships in this context, where the caregivers aim to help the female patients to gain autonomy (even during the care), better health (overall), and life in society. However, it may be challenging to have a caring relationship if they also need to dictate some of the living conditions for the female patients. The care, caregivers, and female patients are affected by and affect several factors, especially each other. It became clear in this study that the surrounding context could shape the women, even though the caregivers tried to acknowledge the female patients’ interests and personalities. To support female patients, caregivers can apply Trauma-Informed Care (TIC), an approach that fosters a safe environment, engages patients in planning necessary changes, and promotes open communication (26). TIC has six principles, aiming to see the person as a whole human being and to incorporate the consequences of history, trauma, and accessibility of care, while adapting to vulnerabilities and health challenges (27). Caregivers in the present study spoke about female patients as less likely to be affected by severe actions (e.g., abortion) and the caregivers related this to the women’s history.

Caregivers needed to help the women maintain or rediscover their identity, both as women and, in some cases, as mothers. A previous study highlighted that women struggled to find a coherent sense of self, an identity, and caregivers sought to help them in this task (28). Caregivers in the present study needed to provide support for female patients to establish or maintain healthy relationships, such as with their families. They were not sure that the female patients’ families constituted a positive relationship, as they had seen negative relationships with families. It can be seen as necessary, but at the same time, difficult for caregivers in this context to adhere to the concept of the family system in certain situations. Hörberg et al. (29) reported the lack of research on this topic, but highlighted the importance of prioritizing the family’s needs. These authors also stated that families are involved when they are considered to act in line with the rehabilitation plan, but that family involvement can be difficult when the patient has also victimized them. This was confirmed in the current study and interpreted as the reason why, in some cases, families do not want contact, or why the patient herself does not desire to have contact. As previously stated, the women’s offences have often been directed towards family and children (4), which could be a reason that caregivers find it challenging to include families, but also to speak to the female patients about their children and family whom they might have committed crimes against. Despite this, the current study revealed that caregivers attempted to foster a relationship between the female patients and their families. Involving families is seen as necessary, but also a result of both the female patients’ and their families’ willingness to be involved in this type of care and the female patients’ lifeworld.

The caregivers did not want to make distinctions based on the patients’ gender, but at the same time, they noted that the care environment and some of their own behaviors were affected by the patients’ gender. In a previous study focusing on female patients, women reported that caregivers wanted them to behave in a certain way, being humble was perceived as disadvantageous, and they felt they needed to be more straightforward and assertive (17). The caregivers in the present study perceived female patients as being less dangerous than male patients, and they sought to calm the women. Another study of female and male patients showed that caregivers tried to create a calming atmosphere by providing a friendly and caring presence as well as instilling faith, comfort, and predictability (9).

The individuals (patients and caregivers) involved in this type of care experience the actions and non-actions in different ways due to having different experiences and backgrounds. They are in the environment for diametrically opposite reasons; one group provides care while the other receives care. The female patients evoke feelings that are difficult to define, and managing these feelings can be challenging. An approach incorporating TIC could be beneficial for caregivers in maintaining resilience and delivering consistent care (26). Although female patients were in the minority, they made a notable impact on the caregivers, and it was easy to relate a new female patient to one who had been cared for before. TIC could also be necessary for caregivers to remain resilient and provide care that is not affected by emotions generated by female patients. According to the participants, these women have historically been associated with self-harming behavior, which could affect the way the caregivers acted in relation to them. Sollid and Kvande (7) highlighted the importance of viewing each patient as an individual and understanding how each person perceives and interprets their history and experiences, a finding also supported in the current study. In the interviews, it was mentioned that the female patients had a history of traumatic and challenging experiences, and a few of the participants talked about how this had an impact on the female patients during the care. It is essential to be aware that different interpretations can arise due to the individuals involved, their prior experiences, and the time, place, and circumstances.

One prominent finding in this study was the caregivers’ experiences of performing coercive measures, in particular restraints, in relation to female patients. According to McKeown et al., coercive practices are globally used in response to violent, aggressive, and other behavior among mental health patients (30). Actions included in coercive measures are physical restraint, forced medication (30), and seclusion (31). However, caregivers and patients might not concur on the perceptions and experiences of what constitutes coercion (31). The caregivers in the present study found restraint to be very difficult and something that evoked complicated feelings, leaving a negative impression. Male caregivers talked of feeling as they committed abuse against the female patients in restraint situations. The caregivers (male and female) also spoke of seeing the female patients as highly vulnerable in these situations, causing the caregivers to be close and supportive. Carrying out coercive measures, as a caregiver, seemed to be difficult. Still, their impact on patients should not be underestimated, despite not being found in a study of the life situation of female FPC patients (17). The reason the women did not refer to this subject may be consistent with the caregivers’ assumptions that the female patients had previously experienced even worse situations. The caregivers in the current study shared their experiences working with vulnerable women and the various challenges they faced. Male caregivers mentioned more often than female caregivers the importance of not being alone with the female patients, often due to reducing the risk of them being in situations where they could be accused of sexual harassment. Male caregivers mentioned that the female caregivers were more exposed to violence from female patients, as an interesting finding (or missing), it was less common that female caregivers spoke about male caregivers being in vulnerable situations.

## 6 Conclusion

Being a caregiver for female patients in this context seemed to create experiences of being necessary for another person, as well as being exposed to violence from the same person. The dual experiences continue with telling or involving stories from your own life, as it is crucial to be present, but not overly close; maintaining a distance without being distant. Caregivers need to be aware of these aspects and the importance of sharing experiences and knowledge with others in the field. Aggressive behavior from female patients differed from that of male patients, where the female patients’ aggression was more directed and involved bodily fluids in interpersonal attacks. It can be challenging for caregivers to manage the emotions that arise from such behavior. Still, it is essential for them to set these aside to establish a caring relationship with the female patients, whom they also feel they need to protect.

## 7 Clinical implications/clinical relevance

The results create a greater understanding of how (and what) the caregivers perceive while providing care to the minority group of female patients in FPC. Increased knowledge could help decision-makers improve care by providing caregivers with support and guidelines to use in their everyday clinical practice. It became clear during the interviews that caregivers need to talk about their experiences of delivering care in FPC, which in some cases include coercive measures affecting not only the female patients but the caregivers as well. It could be interpreted as necessary for caregivers to have a forum where they can speak freely about these experiences and learn from one another. This was also illustrated in a recently published article (7), which described the importance of nurses sharing and reflecting on what they observed in their professional team. In summary, we recommend creating development training and/or guidelines for caregivers, as well as a specific forum where they could share experiences with coworkers under the guidance of a professional conversation leader.

## 8 Limitations

The participants in this study were approached via an email sent to all employees (caregivers) on several wards, after which they contacted the data collector if they were interested in participating in the study. Some caregivers may have declined participation due to scheduling conflicts with their clinical responsibilities. Those who participated in the study may also have been caregivers who had recently experienced challenging situations with female patients and felt a need to discuss these experiences. One should also keep in mind that some caregivers are more naturally engaged, and perhaps they find contributing to a study essential; however, even those who did not participate can have valuable (if less reflected) experiences. The authors had no control over this, and it should therefore be taken into consideration when interpreting the results.

## Data Availability

The datasets presented in this article are not readily available because the questions are of a sensitive nature and the datasets presented in the article will thus remain confidential and not be shared due to ethical considerations. Requests to access the datasets should be directed to jessica.revelj@lnu.se.
